# Tanshinone IIA Exerts Anti-Inflammatory and Immune-Regulating Effects on Vulnerable Atherosclerotic Plaque Partially *via* the TLR4/MyD88/NF-κB Signal Pathway

**DOI:** 10.3389/fphar.2019.00850

**Published:** 2019-07-26

**Authors:** Zhuo Chen, Xiang Gao, Yang Jiao, Yu Qiu, Anlu Wang, Meili Yu, Fangyuan Che, Siming Li, Jing Liu, Jingen Li, He Zhang, Changan Yu, Geng Li, Yanxiang Gao, Lin Pan, Weiliang Sun, Jing Guo, Bingyan Cao, Yilin Zhu, Hao Xu

**Affiliations:** ^1^Xiyuan Hospital, China Academy of Chinese Medical Sciences, Beijing, China; ^2^Internal medicine, Tieying Hospital of Fengtai District, Beijing, China; ^3^Graduate School, Beijing University of Chinese Medicine, Beijing, China; ^4^Beijing First Hospital of Integrated Chinese and Western Medicine, Beijing, China; ^5^Cardiovascular Department, Beijing hospital of Traditional Chinese Medicine Shunyi branch, Beijing, China; ^6^Graduate school, China Academy of Chinese Medical, Beijing, China; ^7^Dongzhimen Hospital, The First Affiliated Hospital of Beijing University of Chinese Medicine, Beijing, China; ^8^China-Japan Friendship Hospital, Beijing, China; ^9^Wangjing Hospital, China Academy of Chinese Medical Sciences, Beijing, China

**Keywords:** Tanshinone IIA, atherosclerosis, anti-inflammatory, immune regulation, TLR4/MyD88/NF-κB

## Abstract

**Background:** Tanshinone IIA (Tan IIA), a lipophilic constituent from *Salvia miltiorrhiza* Bunge, has shown a promising cardioprotective effect including anti-atherosclerosis. This study aims at exploring Tan IIA’s anti-inflammatory and immune-regulating roles in stabilizing vulnerable atherosclerotic plaque in ApoE-deficient (ApoE^−/−^) mice.

**Methods:** Male ApoE^−/−^ mice (6 weeks) were fed with a high-fat diet for 13 weeks and then randomized to the model group (MOD) or Tan IIA groups [high dose: 90 mg/kg/day (HT), moderate dose: 30 mg/kg/day (MT), low dose: 10 mg/kg/day (LT)] or the atorvastatin group (5 mg/kg/day, ATO) for 13 weeks. Male C57BL/6 mice (6 weeks) were fed with ordinary rodent chow as control. The plaque stability was evaluated according to the morphology and composition of aortic atherosclerotic (AS) plaque in H&E staining and Movat staining sections by calculating the area of extracellular lipid, collagenous fiber, and foam cells to the plaque. The expression of the Toll-like receptor 4 (TLR4)/myeloid differentiation factor88 (MyD88)/nuclear factor-kappa B (NF-κB) signal pathway in aorta fractions was determined by immunohistochemistry. Serum levels of blood lipid were measured by turbidimetric inhibition immunoassay. The concentrations of tumor necrosis factor-α (TNF-α) and monocyte chemoattractant protein-1 (MCP-1) were detected by cytometric bead array.

**Results:** Tan IIA stabilized aortic plaque with a striking reduction in the area of extracellular lipid (ATO: 13.15 ± 1.2%, HT: 12.2 ± 1.64%, MT: 13.93 ± 1.59%, MOD: 18.84 ± 1.46%, *P* < 0.05) or foam cells (ATO: 16.05 ± 1.26%, HT: 14.88 ± 1.79%, MT: 16.61 ± 1.47%, MOD: 22.08 ± 1.69%, *P* < 0.05) to the plaque, and an evident increase in content of collagenous fiber (ATO: 16.22 ± 1.91%, HT: 17.58 ± 1.33%, MT: 15.71 ± 2.26%, LT:14.92 ± 1.65%, MOD: 9.61 ± 0.7%, *P* < 0.05) to the plaque than that in the model group, concomitant with down-regulation of the protein expression of TLR4, MyD88, and NF-κB p65, and serum level of MCP-1 and TNF-α in a dose-dependent manner. There were no differences in serum TC, LDL, HDL, or TG levels between ApoE^–/–^ mice and those treated with atorvastatin.

**Conclusions:** These results suggest that Tan IIA could stabilize vulnerable AS plaque in ApoE^−/−^ mice, and this anti-inflammatory and immune-regulating effect may be achieved *via* the TLR4/MyD88/NF-κB signaling pathway.

## Introduction

Atherosclerosis (AS) is the leading cause of cardiovascular disease worldwide. It mainly involves aorta and coronary artery. Previous studies mainly focus on the stenosis of vascular lumen caused by AS lesion. However, AS is no longer regarded as just a simple disease with smooth muscle cell proliferation and lipid accumulation in the arterial wall, since an increased number of inflammatory cells and mediators have been detected in recent years. It is now widely accepted that inflammation and immunity play an important role in AS’s pathological process (Major and Harrison, 2011; Weber and Noels, 2011). The underlying pathogenesis of AS involves imbalanced lipid metabolism and chronic inflammation induced by maladaptive immune response. Moreover, both innate and acquired immune responses mediate all stages of AS inflammation from initiation through progression (Libby et al., 2009; Libby, 2012).

Toll-like receptor 4 (TLR4)/myeloid differentiation factor88 (MyD88)/nuclear factor-kappa B (NF-κB) signal pathway is one of the emerging anti-inflammatory and immune-regulating pathways. TLR4 is the bridge between immune response, chronic inflammation, and lipid metabolism. Activation of TLR4 results in profound consequences on the monocyte aggregation and foam cell formation in AS (Cole et al., 2010). The lack of MyD88 causes a remarkable reduction in plaque area, expression of proinflammatory gene, and secretion of inflammatory cytokines (Björkbacka et al., 2004). NF-κB, located in the downstream of the TLR4 signal pathway, is the key regulator of the TLR4/MyD88/NF-κB signal pathway (Moghimpour et al., 2012). Biological stress stimulates cells and then triggers activation of NF-κB, and activated NF-κB enters the nucleus and regulates expression of various inflammatory cytokines (Pamukcu et al., 2011). Together, the TLR4/MyD88/NF-κB signal pathway involves a variety of pathological processes, like cholesterol metabolism, apoptosis, inflammation, and immune response, and vascular remodeling. It is a critical pathway of AS immune and inflammatory mechanism (Wijnand et al., 2010).

*Salvia miltiorrhiza* Bunge is the root of *S. miltiorrhiza* of Labiatae. Tanshinone IIA (Tan IIA), a promising natural cardioprotective agent with immunomodulatory effect, is a lipophilic constituent of *S. miltiorrhiza* Bunge. Previous studies showed that Tan IIA could inhibit formation of AS plaque (Wang et al., 2017), and the mechanism involves endothelial cell protection, anti-oxidation, anti-inflammation, and anti-platelet aggregation (Shang et al., 2012). Recent studies showed that Tan IIA plays an anti-inflammatory and immunomodulatory role in anti-AS by inhibiting dendritic cell-mediated acquired immunity (Kim et al., 2012; Li et al., 2014). This makes Tan IIA a promising drug candidate in anti-inflammatory and immune therapy of AS. Our previous study showed that *S. miltiorrhiza* Bunge could significantly reduce the ratio of lipid to collagen composition and inhibit expression of granulocyte-macrophage colony-stimulating factor (GM-CSF) and NF-κB in aortic plaque of ApoE^−/−^ mice, and thus plays a role in stabilizing vulnerable plaque (Zhou et al., 2008). Recently, we found that Tan IIA significantly reduced levels of serum inflammatory cytokines, such as high-sensitivity C-reactive protein (hs-CRP), interleukin-6 (IL-6), and monocyte chemoattractant protein-1 (MCP-1) in patients with unstable angina/non-ST segment elevation myocardial infarction (Shang et al., 2013; Li et al., 2017). Nevertheless, it has not been soundly established whether the TLR4/MyD88/NF-κB signal pathway is involved in Tan IIA’s inhibition of inflammation and immune in AS. This study aims at investigating whether Tan IIA exerts anti-inflammatory and immune-regulating roles in stabilizing vulnerable AS plaque in ApoE^−/−^ mice *via* the TLR4/MyD88/NF-κB signaling pathway.

## Materials and Methods

### Animal Models

Male ApoE^−/−^ mice (*n* = 64, 6 weeks old) and male C57BL/6 mice (*n* = 10, 6 weeks old) (Beijing HFK Bioscience Co. Ltd) were allowed to acclimatize with standard laboratory diet for 1 week. The ApoE^−/−^ mice were then fed with a high-fat diet [fat: 21% (wt/wt), cholesterol: 0.15% (wt/wt)] and C57BL/6 mice were fed with ordinary rodent chow (Vital River Laboratory Animal Technology Co. Ltd) for 13 weeks. After 13 weeks, four ApoE^−/−^ mice were randomly selected and sacrificed. Aortic roots were taken under sterile conditions. Degree of AS was evaluated by hematoxylin–eosin (H&E) staining. The rest of the 60 ApoE^−/−^ mice were randomly divided into five groups (*n* = 12): model group (MOD: high-fat diet), high dose of Tan IIA group (HT: high-fat diet and Tan IIA 90 mg/kg/day), moderate dose of Tan IIA group (MT: high-fat diet and Tan IIA 30 mg/kg/day), low dose of TanIIA group (LT: high-fat diet and Tan IIA 10 mg/kg/day), and atorvastatin group (ATO: high-fat diet and atorvastatin 5 mg/kg/day) (Tan IIA standard sample was purchased from Nanjing Zelang pharmaceutical technology Co., Ltd, lot number: ZL140508582; atorvastatin was purchased from Pfizer Pharmaceutical Co., Ltd, lot number: 046012K). Ten C57BL/6 mice composed control group (CON: fed with ordinary rodent chow). Each group was administered the same amount of 0.5% sodium carboxymethyl cellulose solution (CMC-Na) to mix drugs (CON and MOD group with no drug) by gavage once a day simultaneously.

### “En face’’ Observation of Arterial Tree: Sudan IV Staining

After perfusion fixation, aortas were opened longitudinally from brachiocephalic artery to iliac artery bifurcation. Aortas were fixed to a pan with peripheral fat and adventitial tissue removed under a dissecting microscope. The aortas were then stained with filtered Sudan IV working solution (0.5 g Sudan IV, 50 ml acetone, 50 ml 70% ethyl alcohol) for 6 min and then destained for 5 min in 80% ethyl alcohol. The images were captured with a digital color camera (Canon IXUS 110 IS).

### Histopathological Staining (H&E Staining and Movat Staining) and Immunohistochemistry

The aortic roots were made into 4-μm consecutive paraffin sections of the following four surfaces (Suzuki et al., 1997): 1) the transverse section of ascending aorta root, 2) the aortic valve attachment section, 3) the beginning part of the aortic valve, and 4) the aortic valve fusion aspect. The main dyes of Movat staining set were biebrich scarlet, picric acid, and saffron essence. H&E and Movat stainings were prepared and carried out as operation procedure described before (Tangirala et al., 1995; Bea et al., 2002). H&E staining was used to examine extracellular lipid components (cholesterol crystals and cholesterol ester) and basic lesion morphology. Movat staining was used to examine foam cells, collagen, and buried fibrous cap in plaque. Immunohistochemistry was performed by Envision method. (Sabattini E et al.,1998)

Paraffin sections was dewaxed and blocked with endogenous peroxidase by 3% H_2_O_2_, followed by incubation with a rabbit polyclonal antibody against TLR4, MyD88, and NF-κB p65 (Abcam, Cambridge, MA) at 4°C overnight. After washing, the sections were incubated with an Fluorescein (FITC)-labeled secondary goat anti-rabbit antibody for 1 h. Antigens were visualized with diaminobenzidine (DAB). Immunostained paraffin sections were photographed on a light microscope (Olympus, B×51, Tokyo, Japan) and a proportion of positive immunostained sections were analyzed using Image-Pro Plus 6.0.

### Measurement of Serum Level of Blood Lipid

The serum was separated from blood samples that were collected by removing eyeballs. The concentrations of total cholesterol (TC), high-density lipoprotein (HDL), low-density lipoprotein (LDL), and triglyceride (TG) were determined using an automatic biochemical analyzer (RX-2000, Technicon, USA). TC and TG were measured by enzymatic method; LDL and HDL were measured by turbidimetric inhibition immunoassay.

### Measurement of Serum Level of Inflammatory Factors

The levels of cytokines (TNF-α, MCP-1) were detected by using cytometric bead array (CBA) mouse inflammation kit according to the manufacturer’s instructions (BD PharMingen, San Diego, CA, USA).

### Statistical Analysis

All data are presented as mean ± standard error of mean (SEM). A value of *P* < 0.05 was considered statistically significant. One-way analysis of variance (ANOVA) was adopted followed by LSD *post hoc* analysis for multiple comparisons. Statistical analyses were performed using the SPSS 19.0 software (SPSS Inc, Chicago, IL, USA).

## Results

### Confirmation of AS Formation in ApoE^−/−^ Mice After a High-Fat Diet for 13 Weeks

**Figure 1:**
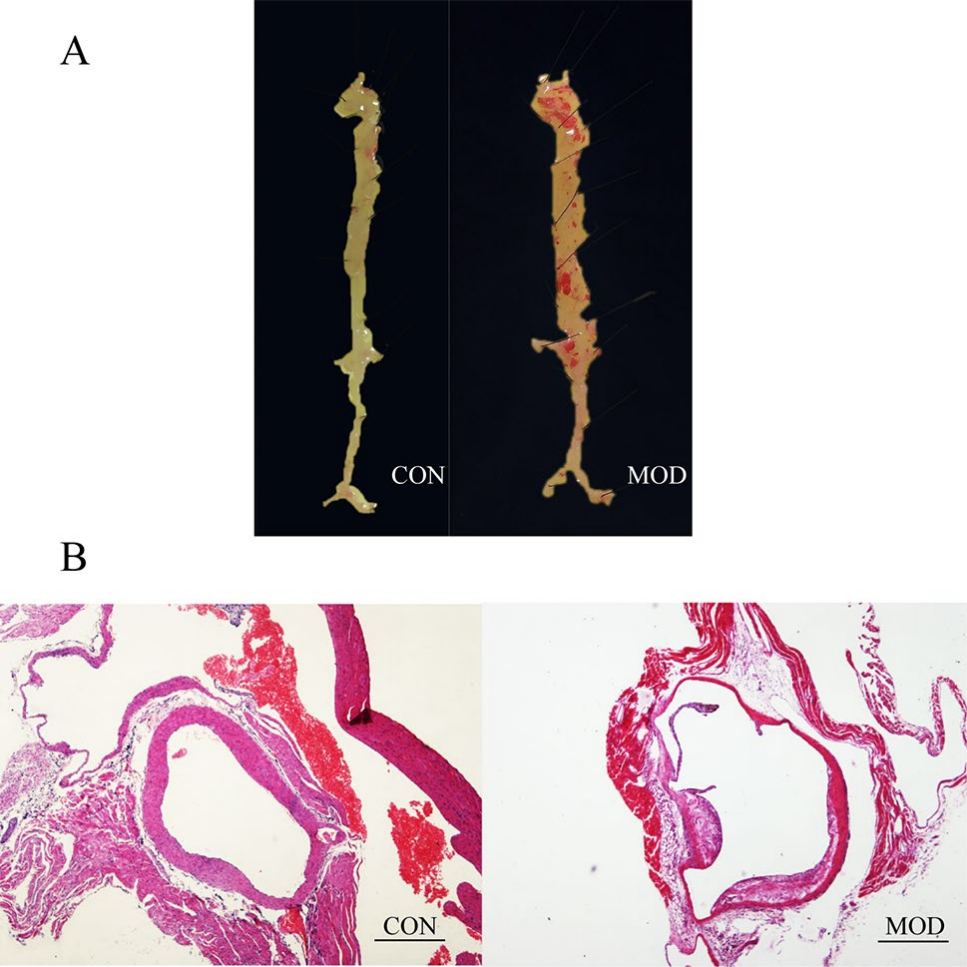
**(A)** Atherosclerotic (AS) formation of ApoE^−/−^ mice after a high-fat diet for 13 weeks compared with C57BL/6 mice by “En face” observation. **(B)** AS formation of ApoE^−/−^ mice after a high-fat diet for 13 weeks compared with C57BL/6 mice by hematoxylin–eosin (H&E) staining on light microscope. CON: C57BL/6 mice, MOD: ApoE^−/−^ mice (bar = 500 µm).

He staining was applied on aortic roots of ApoE^−/−^ mice and C57BL/6 mice to identify AS. “En face” observation showed distinct dyed red plaque in the arterial tree of ApoE^−/−^ mice and smooth arterial vessels in C57BL/6 mice in contrast (**Figure 1A**). Furthermore, plaque with obvious lipid core and foam cells that infiltrated into the extima of aorta were observed in the ApoE^−/−^ mice’s aortas, compared with that of C57BL/6 mice after H&E staining on a light microscope (**Figure 1B**). Hence, it was identified that AS was developed in ApoE^−/−^ mice after a high-fat diet for 13 weeks.

### Tan IIA Reduced AS Severity and Stabilized Plaque

**Figure 2:**
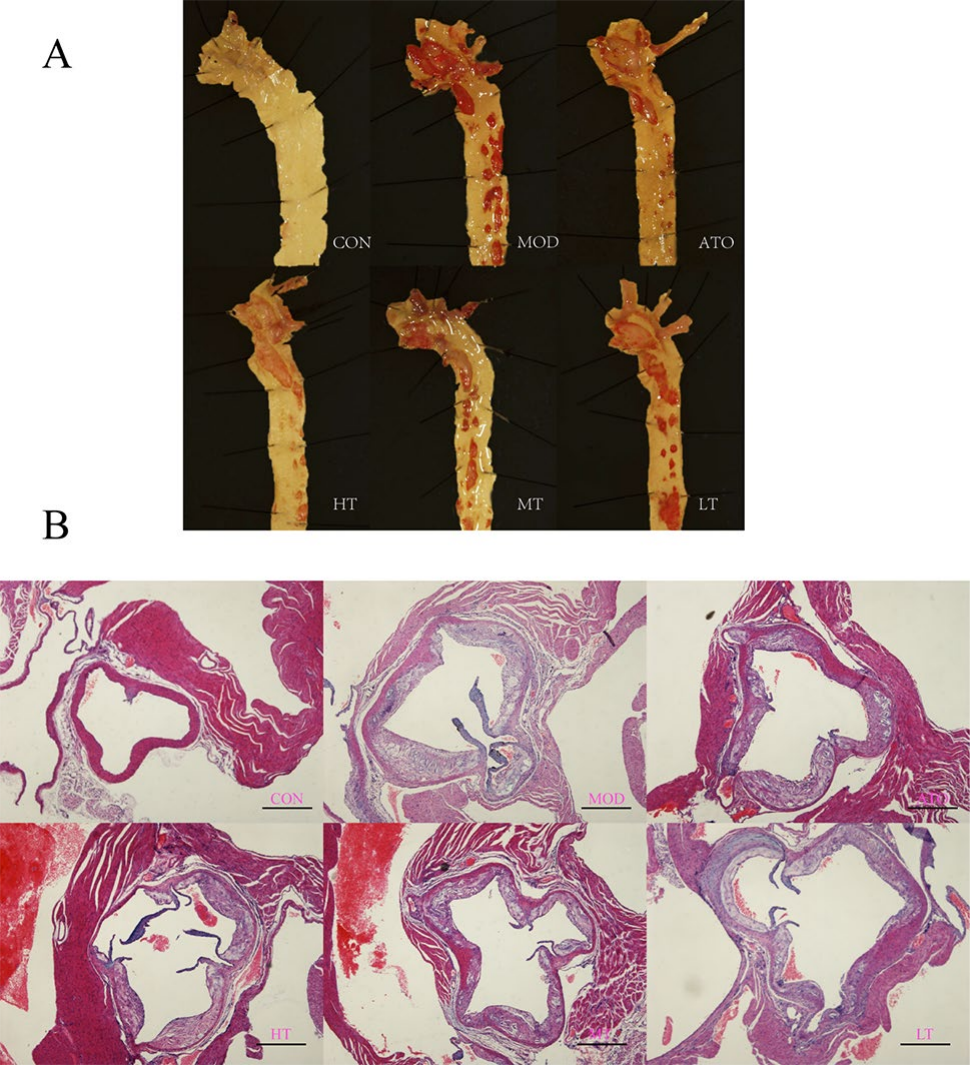
Tan IIA and atorvastatin attenuated degree of AS lesion in ApoE^−/−^ mice. **(A)** “En face” observation of aortic roots. **(B)** H&E staining of aortic roots. A: CON, B: MOD, C: ATO, D: HT, E: MT, F: LT (bar = 500 µm).

Firstly, two ApoE^−/−^ mice in each group and one in control group were selected randomly for “En face” observation of aortic arch and thoracic aorta. It was intuitively plausible that the aorta of the CON group was smooth and clean, that of MOD group had the most plaque, and other groups using either Tan IIA or Atorvastatin had less plaque than the MOD group to some extent (**Figure 2A**).

**Figure 3:**
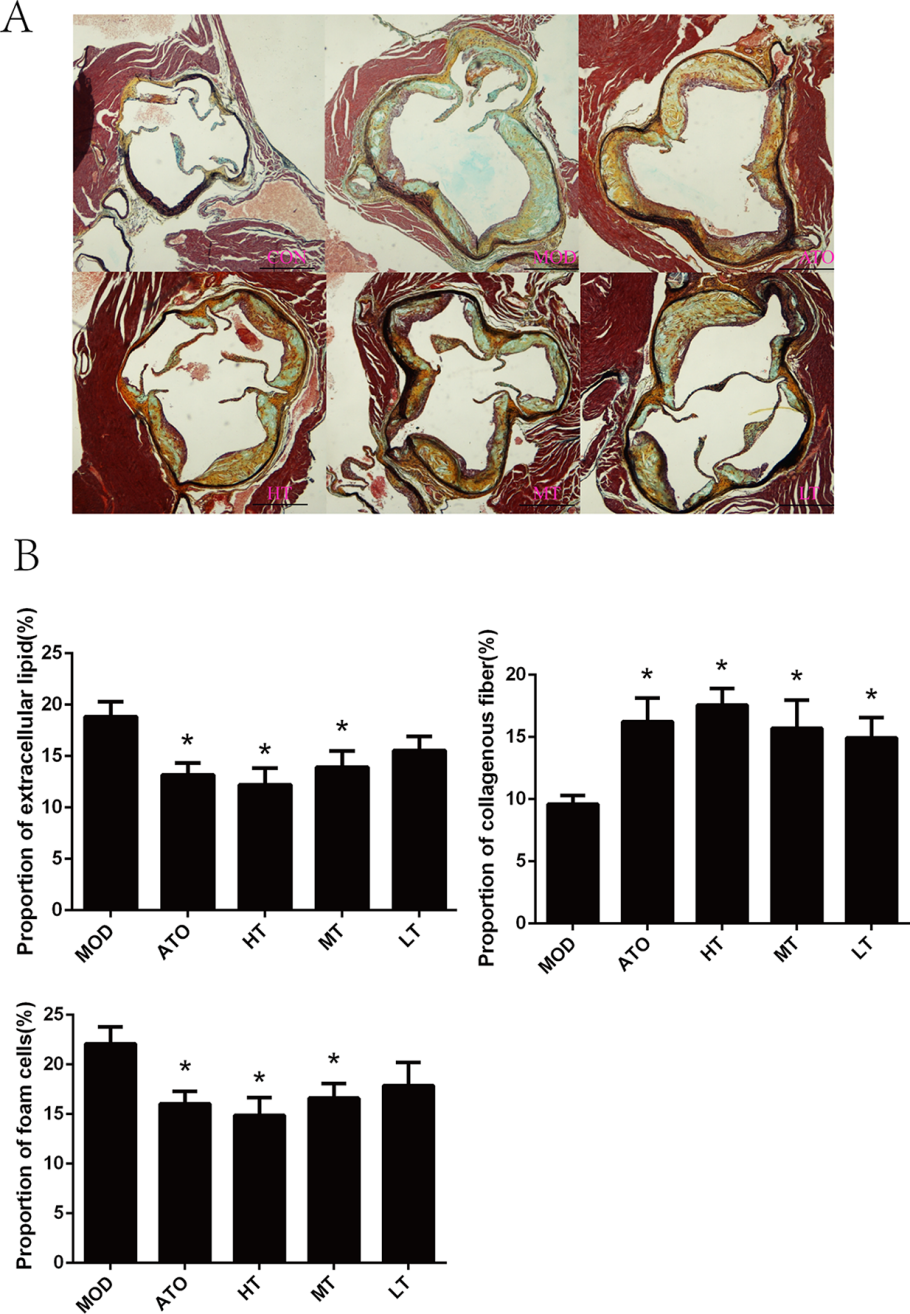
**(A)** Movat staining of aortic roots and **(B)** the proportion of extracellular lipid, collagenous fiber and foam cells to plaque. Data are expressed as mean ± SEM. n = 12 (except CON group, n = 9). *P < 0.05 versus MOD group. A: CON, B: MOD, C: ATO, D: HT, E: MT, F: LT (bar = 500 µm).

Further observation of H&E and Movat staining of aortic roots were shown respectively in **Figures 2B** and **3A**. We found that the ratio of extracellular lipid to plaque was significantly reduced in ApoE^−/−^ mice treated with ATO, HT, and MT (ATO: 13.15 ± 1.2%, HT: 12.2 ± 1.64%, MT: 13.93 ± 1.59%) (*P* < 0.05 versus MOD: 18.84 ± 1.46%) (**Figure 3B**). The proportion of collagen fiber to plaque in the ATO group and all Tan IIA groups were higher than that in the MOD group (*P* < 0.05) (**Figure 3B**) (ATO: 16.22 ± 1.91%, HT: 17.58 ± 1.33%, MT: 15.71 ± 2.26%, LT: 14.92 ± 1.65%, MOD: 9.61 ± 0.7%). A dose-dependent relationship was found between different dose groups of Tan IIA. These results indicated that both atorvastatin and Tan IIA could reduce extracellular lipid and increase collagen fiber so as to make plaques stable.

### TanIIA Decreased the Blood Lipid in ApoE^–/–^ Mice

As expected, a marked increase was seen in the MOD group compared with the CON group in TC, LDL, and HDL (*P* < 0.05). Tan IIA reduced TC in a dose-dependent manner (*P* < 0.05). HT and MT groups lowered LDL (*P* < 0.05). A statistically significant increase in HDL was only observed in the LT group (*P* < 0.05). In general, AS is mostly associated with LDL and TC. There was no statistical difference found in TG between MON and CON. However, there were no differences in serum TC, LDL, HDL, or TG levels between ApoE^–/–^ mice and those treated with atorvastatin (**Table 1**).

**Table 1:** Blood lipid profile of each group.

Group	N	TC(mmol/L)	LDL(mmol/L)	HDL(mmol/L)	TG(mmol/L)
CON	9	2.52±0.08	0.56±0.02	2.64±0.10	0.41±0.10
MOD	12	25.58±1.55^#^	18.74±1.18^#^	10.86±0.70^#^	0.65±0.09
ATO	12	23.36±1.22	17.32±0.95	9.78±0.54	0.52±0.11
HT	12	19.42±1.05*	13.97±0.80*	9.08±0.48	0.55±0.12
MT	12	21.10±1.79*	15.55±1.28*	9.32±0.69	0.41±0.07*
LT	12	21.50±1.58*	15.94±1.21	15.94±1.21*	0.46±0.06

Data are expressed as mean ± SEM. ^#^P < 0.05 vs CON group; *P < 0.05 vs MOD group.

### TanIIA Inhibited Vascular Inflammation in ApoE^–/–^ Mice

The degree of macrophage adhesion and infiltration, shown as the proportion of foam cells to plaque, was selected to determine the anti-inflammatory effect of Tan IIA on aorta. Macrophage phagocytosed lots of oxidized low-density lipoprotein (ox-LDL), leading to lipid accumulation within the cells, and finally caused the formation of foam cells. A reduction of foam cell content in atherosclerotic lesions was observed in atorvastatin or Tan IIA-treated groups compared with ApoE^–/–^ mice by Movat staining (*P* < 0.05, ATO: 16.05 ± 1.26%, HT: 14.88 ± 1.79%, MT: 16.61 ± 1.47%, MOD: 22.08 ± 1.69%) (**Figure 3B**).

**Figure 4:**
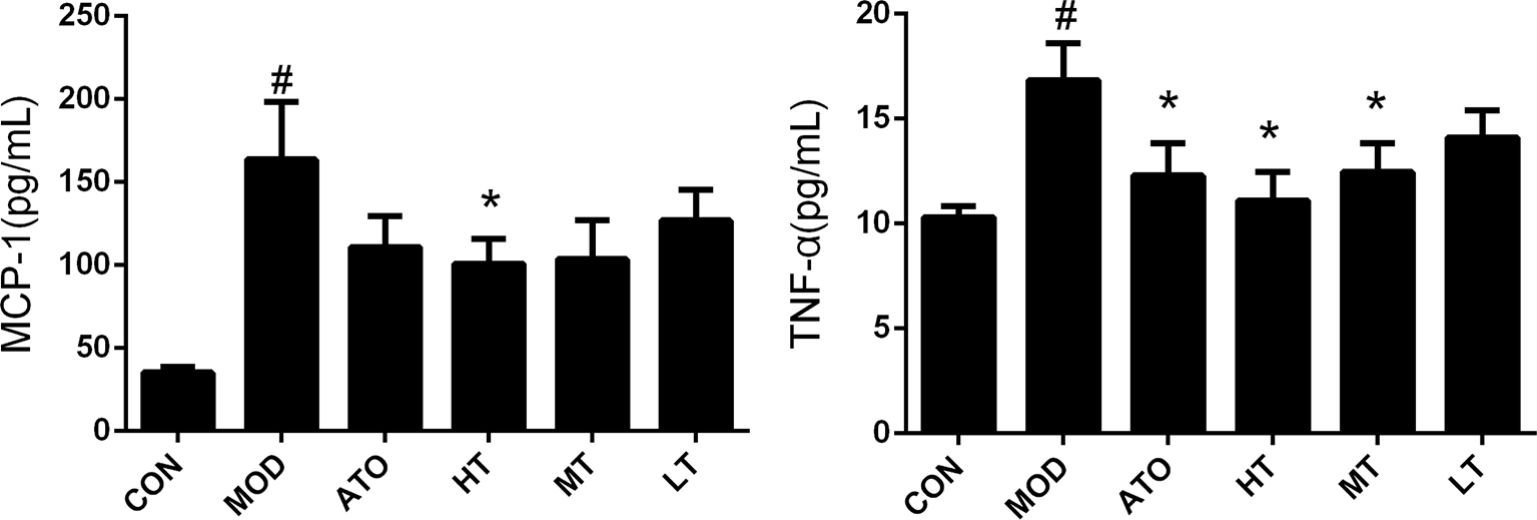
Effect of TanIIA on the level of monocyte chemoattractant protein-1 (MCP-1) and tumor necrosis factor-α (TNF-α) in ApoE^−/−^ mice. Data are expressed as mean ± SEM. *n* = 12 (except CON group, *n* = 9). *P < 0.05 versus MOD group. ^#^ *P* < 0.05 versus CON group.

In addition, the concentration of MCP-1 was elevated in the MOD group, compared to the CON group by cytometric bead array (163.75 ± 34.53 pg/ml vs 35.39 ± 3.51 pg/ml, *P* < 0.05), and reduced in the HT group, compared to the MOD group (100.94 ± 15.09 pg/ml vs 163.75 ± 34.53 pg/ml, *P* < 0.05) (**Figure 4**). The concentration of TNF-α was elevated in the MOD group compared to the CON group (16.84 ± 1.75 pg/ml vs 10.3 ± 0.54 pg/ml, *P* < 0.05), and decreased by Tan IIA (HT: 11.12 ± 1.35 pg/ml, MT: 12.46 ± 1.39 pg/ml, *P* < 0.05) and atorvastatin (12.29 ± 1.55 pg/ml, *P* < 0.05) intervention in reverse (**Figure 4**). These findings indicated that treatment with TanIIA could inhibit AS inflammation.

### Tan IIA Regulated Expression of the TLR4/MyD88/NF-κB Signal Pathway in Aorta

**Figure 5:**
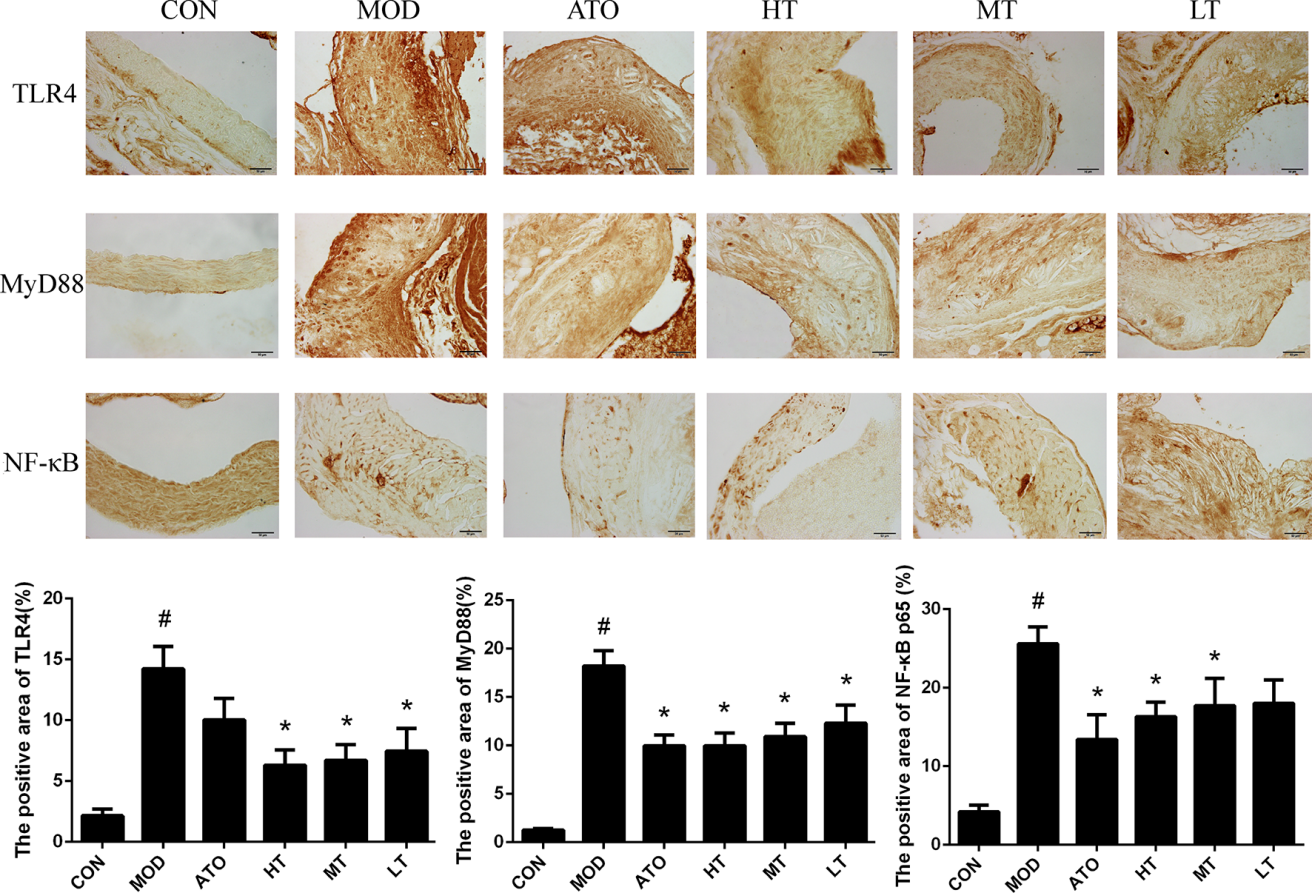
Effects of Tan IIA treatment on the expression of TLR4, MyD88, and nuclear factor-kappa B (NF-κB) in plaque in aortic root of ApoE^−/−^ mice by immunohistochemistry and the positive area of TLR4, MyD88, and NF-κB p65 in plaque by immunohistochemistry. Data are expressed as mean ± SEM. *n* = 12 (except CON group, *n* = 9). *P < 0.05 versus MOD group; ^#^ *P* < 0.05 versus CON group.

In order to elucidate the molecular mechanism underlying Tan IIA’s anti-inflammation effect, we evaluated the expression of TLR4, MyD88, and NF-κB p65 in aorta fractions by immunohistochemistry (**Figure 5**). The levels of TLR4, MyD88, and NF-κB p65 in the MOD group were all higher than those of the CON group (*P* < 0.05). Tan IIA treatment down-regulated expression of TLR4 compared with the MOD group in a dose-dependent manner (MOD: 14.23 ± 1.84%, HT: 6.32 ± 1.26%, MT: 6.71 ± 1.28%, LT: 7.46 ± 1.88%) (*P* < 0.05). Both ATO and all Tan IIA treatment groups decreased expression of MyD88 (MOD: 18.23 ± 1.55%, ATO: 9.97 ± 1.12%, HT: 9.98 ± 1.32%, MT: 10.9 ± 1.39%, LT: 12.29 ± 1.86%) and NF-κB p65 (MOD: 25.59 ± 2.15%, ATO: 13.41 ± 3.14%, HT: 16.31 ± 1.85%, MT: 17.68 ± 3.53%), compared with the MOD group (*P* < 0.05) (**Figure 5**).

## Discussion

Our data strongly supported that Tan IIA was a new drug candidate for inhibiting inflammation and regulating immunity in AS. In our study, we found that AS was formed initially in ApoE^−/−^ mice fed with a high-fat diet for 13 weeks and reached advanced stage after 26 weeks. Expressions of inflammatory factors and key proteins in the TLR4/MyD88/NF-κB signal pathway were increased in the ApoE^−/−^ mice group compared with the C57BL/6 mice group. The data presented here suggested that Tan IIA stabilizes plaque by reducing the inflammatory and immune response in a dose-dependent manner.

Whether the plaque is stable or not is determined by the proportion of extracellular lipid, collagen, macrophages, or smooth muscle cells. In our study, we found obvious AS plaque, foam cells, lipid core, and thin fibrous cap in the model group after 26 weeks. In other words, vulnerable plaque was formed in the model group with local macrophage accumulation, severely damaged vascular membrane, lipid erosion, and calcification. We found that Tan IIA reduced ratio of extracellular lipid and macrophage infiltration and increased the proportion of collagen and therefore stabilized AS plaque. Our findings are consistent with previous findings (Fang et al., 2007).

ApoE^−/−^ mice fed a high-fat diet is a well-established model that was used in our previous study to research AS (Zhou et al., 2008). Stability of plaque is determined by content of extracellular lipid, macrophage, and collagen (Libby et al., 2002). Here, our study found that Tan IIA lowered extracellular lipid and macrophage infiltration and increased proportion of collagen and therefore stabilized AS lesions. This is consistent with previous findings (Tang et al., 2011; Xu et al., 2011).

Statins did not decrease serum lipid in ApoE^−/−^ mice, because they lack a critical ligand (ApoE) for the LDL receptor that is important for lowering cholesterol (Sorrentino and Landmesser, 2005; Zadelaar et al., 2007; Zhou and Liao, 2010). However, whether Tan IIA has effects on serum levels of lipids is still not clear. Some former studies’ improved Tan IIA had no effect on serum lipid profiles (Tang et al., 2007; Chen et al., 2012). On the contrary, we demonstrated that Tan IIA lowered LDL, TC, and TG. This is not surprising because Tan IIA is equipped with biological pleiotropic effects. Lipid metabolism is the key link of AS, but AS is a complex and comprehensive disease, so we speculated that merely lowering serum lipid is insufficient to elucidate the inhibitory effect of Tan IIA on AS.

AS was first regarded as an autoimmune disease in 1992 (Wick G et al,1992). It is also a chronic inflammatory disorder involving both the innate and adaptive immune response (Packard et al., 2009; Grundtman and Wick, 2011). Recently, Tan IIA is confirmed as a promising natural cardioprotective agent with anti-inflammatory and immunomodulatory effects on AS (Chen and Xu, 2014). Tan IIA could inhibit AS accompanied with repression of inflammatory cytokines such as VCAM-1, ICAM-1 (Chang et al., 2014), P-selectin (Jiang et al., 1998), MMP-2, MMP-9, IL-1β (Fang et al., 2007), and IL-10 (Guo et al., 2009). Among them, MCP-1 is a kind of chemokine that induces monocyte adhesion to endothelial cells. Activation of TNF-α is an important step of the inflammatory cascade reaction. ICAM-1 gathers leukocyte in certain parts, inducing local tissue injury and inflammation. In our study, it was demonstrated that MCP-1, TNF-α, and ICAM-1 dramatically increased in the model group and reversely decreased in Tan IIA groups. Similar to previous studies (Wang et al., 2017), our data strongly verified the anti-inflammatory effect of Tan IIA on AS.

The TLR4 signal pathway associated with inflammation and immunity has been uncovered in many diseases, such as intracerebral hemorrhage (Lin et al., 2012), osteoarthritis (Liu et al., 2014), renal fibrosis, and chronic kidney disease (Souza et al., 2015). A growing body of evidence suggests that the TLR4 signal pathway might also be involved in the inflammation and immune response during AS (Edfeldt et al., 2002; Shinohara et al., 2007). From an innate immunity perspective, the most likely mechanism of AS is activation of Toll-like receptors or MyD88 (Björkbacka et al., 2004; Michelsen et al., 2004). NF-κB is the key factor to trigger downstream inflammation. Tan IIA could play a suppressive role in AS by virtue of inhibition of the I-kappaB kinase (IKK)/NF-κB signal pathway that was activated by TNF-α, accompanied by repression of chemokines. One study showed that Tan IIA inhibited lipopolysaccharide (LPS)-induced inflammation and reduced expression of TLR4 and TNF-α (Jia et al., 2001). Based on these findings, we further revealed that expression of TLR4, MyD88, and NF-κB was elevated in the aorta of ApoE^−/−^ mice compared with that of C57BL/6 mice. We demonstrated, for the first time, that Tan IIA played an important role in stabilizing atherosclerotic plaque by downregulating the key protein expression in the TLR4/MyD88/NF-κB pathway. Previous studies mostly focused on certain gene expression but ignored the signal pathway and its upstream- and downstream-related factors. Our present study established the mechanism that Tan IIA inhibited AS immune and inflammation, partly through the TLR4/MyD88/NF-κB signal pathway.

AS is a chronic disease that requires long-term treatment, merely inhibition of TLR4/NF-κB may cause other potential adverse effects (such as infection and cancer) or other complications. Therefore, it is necessary to explore the role of TLRs in normal physiological conditions and their interaction with other factors in the immune-inflammatory network, as well as the possible consequences of blocking TLRs on many different types of cells. There is no doubt that the anti-inflammatory and immunomodulatory effects of Tan IIA provide a new candidate for the prevention and treatment of AS.

## Ethics Statement

All experiments were conducted in accordance with the Guide for Care and Use of Laboratory Animals as adopted and promulgated by the United National Institutes of Health. All experimental protocols were approved by the Review Committee for the Use of Animal Subjects of Xiyuan Hospital.

## Author Contributions

HX and YQ designed experiments. ZC, XG, YJ, AW, MY, CY, GL, YG, LP, WS, and JG carried out experiments. ZC, FC, JingL, JingeL, and HZ analyzed experimental results. ZC, SL, BC, and YZ wrote the manuscript.

## Funding

The authors declare that there is no conflict of interests regarding the publication of this paper. This work is supported by grants from the National Natural Science Foundation of China (No. 81373823) and Youth foundation of the national natural science foundation of China (No.81703928).

## Conflict of Interest Statement

The authors declare that the research was conducted in the absence of an.y commercial or financial relationships that could be construed as a potential conflict of interest.

## Abbreviations

Tan IIA, Tanshinone IIA; TLR4, Toll-like receptor 4; MyD88, myeloid differentiation factor88; NF-κB, nuclear factor-kappa β; TNF-α, tumor necrosis factor-α; MCP-1, monocyte chemoattractant protein-1; LPS, lipopolysaccharide; AS, atherosclerosis; GM-CSF, granulocyte-macrophage colony-stimulating factor; hs-CRP, high-sensitivity C-reactive protein; IL-6, interleukin-6; H&E hematoxylin–eosin; HT, high dose of Tan IIA group, MT, middle dose of Tan IIA group, LT, low dose of Tan IIA group; MON, model group; CON, control group; ox-LDL, oxidized low-density lipoprotein.
